# Supplementary material for: The effects of genital myiasis on the diversity of the vaginal microbiota in female Bactrian camels
Source: BMC Vet Res. 2022 Mar 5;18:87. doi: 10.1186/s12917-022-03189-5 (PMC8897907; doi:10.1186/s12917-022-03189-5)
Supplement: Supplementary file 5 — Additional file 5. [file 12917_2022_3189_MOESM5_ESM.zip › MPL201709200_16s_yy/Treat1/css/js_css/jsPDF-master.zip!/jsPDF-master/docs/test_libs_qunit.js.html]

test/libs/qunit.js - Documentation


## Home

### Classes

- FontObject
- jsPDF
- PubSub

### Global

- addFont
- addMetadata
- addPage
- CapJoinStyles
- circle
- CssColors
- ellipse
- getFontList
- html2pdf
- lines
- output
- rect
- requirejs
- reset
- roundedRect
- save
- setDrawColor
- setFillColor
- setFont
- setFontSize
- setFontStyle
- setLineCap
- setLineJoin
- setLineWidth
- setProperties
- setTextColor
- text
- toUrl
- triangle
- triggerEvent

# test/libs/qunit.js

```
/**
 * QUnit v1.3.0pre - A JavaScript Unit Testing Framework
 *
 * http://docs.jquery.com/QUnit
 *
 * Copyright (c) 2012 John Resig, Jörn Zaefferer
 * Dual licensed under the MIT (MIT-LICENSE.txt)
 * or GPL (GPL-LICENSE.txt) licenses.
 */

(function(window) {

var defined = {
	setTimeout: typeof window.setTimeout !== "undefined",
	sessionStorage: (function() {
		var x = "qunit-test-string";
		try {
			sessionStorage.setItem(x, x);
			sessionStorage.removeItem(x);
			return true;
		} catch(e) {
			return false;
		}
	})()
};

var	testId = 0,
	toString = Object.prototype.toString,
	hasOwn = Object.prototype.hasOwnProperty;

var Test = function(name, testName, expected, async, callback) {
	this.name = name;
	this.testName = testName;
	this.expected = expected;
	this.async = async;
	this.callback = callback;
	this.assertions = [];
};
Test.prototype = {
	init: function() {
		var tests = id("qunit-tests");
		if (tests) {
			var b = document.createElement("strong");
				b.innerHTML = "Running " + this.name;
			var li = document.createElement("li");
				li.appendChild( b );
				li.className = "running";
				li.id = this.id = "test-output" + testId++;
			tests.appendChild( li );
		}
	},
	setup: function() {
		if (this.module != config.previousModule) {
			if ( config.previousModule ) {
				runLoggingCallbacks('moduleDone', QUnit, {
					name: config.previousModule,
					failed: config.moduleStats.bad,
					passed: config.moduleStats.all - config.moduleStats.bad,
					total: config.moduleStats.all
				} );
			}
			config.previousModule = this.module;
			config.moduleStats = { all: 0, bad: 0 };
			runLoggingCallbacks( 'moduleStart', QUnit, {
				name: this.module
			} );
		} else if (config.autorun) {
			runLoggingCallbacks( 'moduleStart', QUnit, {
				name: this.module
			} );
		}

		config.current = this;
		this.testEnvironment = extend({
			setup: function() {},
			teardown: function() {}
		}, this.moduleTestEnvironment);

		runLoggingCallbacks( 'testStart', QUnit, {
			name: this.testName,
			module: this.module
		});

		// allow utility functions to access the current test environment
		// TODO why??
		QUnit.current_testEnvironment = this.testEnvironment;

		try {
			if ( !config.pollution ) {
				saveGlobal();
			}

			this.testEnvironment.setup.call(this.testEnvironment);
		} catch(e) {
			QUnit.ok( false, "Setup failed on " + this.testName + ": " + e.message );
		}
	},
	run: function() {
		config.current = this;
		if ( this.async ) {
			QUnit.stop();
		}

		if ( config.notrycatch ) {
			this.callback.call(this.testEnvironment);
			return;
		}
		try {
			this.callback.call(this.testEnvironment);
		} catch(e) {
			fail("Test " + this.testName + " died, exception and test follows", e, this.callback);
			QUnit.ok( false, "Died on test #" + (this.assertions.length + 1) + ": " + e.message + " - " + QUnit.jsDump.parse(e) );
			// else next test will carry the responsibility
			saveGlobal();

			// Restart the tests if they're blocking
			if ( config.blocking ) {
				QUnit.start();
			}
		}
	},
	teardown: function() {
		config.current = this;
		try {
			this.testEnvironment.teardown.call(this.testEnvironment);
			checkPollution();
		} catch(e) {
			QUnit.ok( false, "Teardown failed on " + this.testName + ": " + e.message );
		}
	},
	finish: function() {
		config.current = this;
		if ( this.expected != null && this.expected != this.assertions.length ) {
			QUnit.ok( false, "Expected " + this.expected + " assertions, but " + this.assertions.length + " were run" );
		}

		var good = 0, bad = 0,
			tests = id("qunit-tests");

		config.stats.all += this.assertions.length;
		config.moduleStats.all += this.assertions.length;

		if ( tests ) {
			var ol = document.createElement("ol");

			for ( var i = 0; i < this.assertions.length; i++ ) {
				var assertion = this.assertions[i];

				var li = document.createElement("li");
				li.className = assertion.result ? "pass" : "fail";
				li.innerHTML = assertion.message || (assertion.result ? "okay" : "failed");
				ol.appendChild( li );

				if ( assertion.result ) {
					good++;
				} else {
					bad++;
					config.stats.bad++;
					config.moduleStats.bad++;
				}
			}

			// store result when possible
			if ( QUnit.config.reorder && defined.sessionStorage ) {
				if (bad) {
					sessionStorage.setItem("qunit-" + this.module + "-" + this.testName, bad);
				} else {
					sessionStorage.removeItem("qunit-" + this.module + "-" + this.testName);
				}
			}

			if (bad == 0) {
				ol.style.display = "none";
			}

			var b = document.createElement("strong");
			b.innerHTML = this.name + " <b class='counts'>(<b class='failed'>" + bad + "</b>, <b class='passed'>" + good + "</b>, " + this.assertions.length + ")</b>";

			var a = document.createElement("a");
			a.innerHTML = "Rerun";
			a.href = QUnit.url({ filter: getText([b]).replace(/\([^)]+\)$/, "").replace(/(^\s*|\s*$)/g, "") });

			addEvent(b, "click", function() {
				var next = b.nextSibling.nextSibling,
					display = next.style.display;
				next.style.display = display === "none" ? "block" : "none";
			});

			addEvent(b, "dblclick", function(e) {
				var target = e && e.target ? e.target : window.event.srcElement;
				if ( target.nodeName.toLowerCase() == "span" || target.nodeName.toLowerCase() == "b" ) {
					target = target.parentNode;
				}
				if ( window.location && target.nodeName.toLowerCase() === "strong" ) {
					window.location = QUnit.url({ filter: getText([target]).replace(/\([^)]+\)$/, "").replace(/(^\s*|\s*$)/g, "") });
				}
			});

			var li = id(this.id);
			li.className = bad ? "fail" : "pass";
			li.removeChild( li.firstChild );
			li.appendChild( b );
			li.appendChild( a );
			li.appendChild( ol );

		} else {
			for ( var i = 0; i < this.assertions.length; i++ ) {
				if ( !this.assertions[i].result ) {
					bad++;
					config.stats.bad++;
					config.moduleStats.bad++;
				}
			}
		}

		try {
			QUnit.reset();
		} catch(e) {
			fail("reset() failed, following Test " + this.testName + ", exception and reset fn follows", e, QUnit.reset);
		}

		runLoggingCallbacks( 'testDone', QUnit, {
			name: this.testName,
			module: this.module,
			failed: bad,
			passed: this.assertions.length - bad,
			total: this.assertions.length
		} );
	},

	queue: function() {
		var test = this;
		synchronize(function() {
			test.init();
		});
		function run() {
			// each of these can by async
			synchronize(function() {
				test.setup();
			});
			synchronize(function() {
				test.run();
			});
			synchronize(function() {
				test.teardown();
			});
			synchronize(function() {
				test.finish();
			});
		}
		// defer when previous test run passed, if storage is available
		var bad = QUnit.config.reorder && defined.sessionStorage && +sessionStorage.getItem("qunit-" + this.module + "-" + this.testName);
		if (bad) {
			run();
		} else {
			synchronize(run, true);
		};
	}

};

var QUnit = {

	// call on start of module test to prepend name to all tests
	module: function(name, testEnvironment) {
		config.currentModule = name;
		config.currentModuleTestEnviroment = testEnvironment;
	},

	asyncTest: function(testName, expected, callback) {
		if ( arguments.length === 2 ) {
			callback = expected;
			expected = null;
		}

		QUnit.test(testName, expected, callback, true);
	},

	test: function(testName, expected, callback, async) {
		var name = '<span class="test-name">' + escapeInnerText(testName) + '</span>';

		if ( arguments.length === 2 ) {
			callback = expected;
			expected = null;
		}

		if ( config.currentModule ) {
			name = '<span class="module-name">' + config.currentModule + "</span>: " + name;
		}

		if ( !validTest(config.currentModule + ": " + testName) ) {
			return;
		}

		var test = new Test(name, testName, expected, async, callback);
		test.module = config.currentModule;
		test.moduleTestEnvironment = config.currentModuleTestEnviroment;
		test.queue();
	},

	/**
	 * Specify the number of expected assertions to gurantee that failed test (no assertions are run at all) don't slip through.
	 */
	expect: function(asserts) {
		config.current.expected = asserts;
	},

	/**
	 * Asserts true.
	 * @example ok( "asdfasdf".length > 5, "There must be at least 5 chars" );
	 */
	ok: function(a, msg) {
		if (!config.current) {
			throw new Error("ok() assertion outside test context, was " + sourceFromStacktrace(2));
		}
		a = !!a;
		var details = {
			result: a,
			message: msg
		};
		msg = escapeInnerText(msg);
		runLoggingCallbacks( 'log', QUnit, details );
		config.current.assertions.push({
			result: a,
			message: msg
		});
	},

	/**
	 * Checks that the first two arguments are equal, with an optional message.
	 * Prints out both actual and expected values.
	 *
	 * Prefered to ok( actual == expected, message )
	 *
	 * @example equal( format("Received {0} bytes.", 2), "Received 2 bytes." );
	 *
	 * @param Object actual
	 * @param Object expected
	 * @param String message (optional)
	 */
	equal: function(actual, expected, message) {
		QUnit.push(expected == actual, actual, expected, message);
	},

	notEqual: function(actual, expected, message) {
		QUnit.push(expected != actual, actual, expected, message);
	},

	deepEqual: function(actual, expected, message) {
		QUnit.push(QUnit.equiv(actual, expected), actual, expected, message);
	},

	notDeepEqual: function(actual, expected, message) {
		QUnit.push(!QUnit.equiv(actual, expected), actual, expected, message);
	},

	strictEqual: function(actual, expected, message) {
		QUnit.push(expected === actual, actual, expected, message);
	},

	notStrictEqual: function(actual, expected, message) {
		QUnit.push(expected !== actual, actual, expected, message);
	},

	raises: function(block, expected, message) {
		var actual, ok = false;

		if (typeof expected === 'string') {
			message = expected;
			expected = null;
		}

		try {
			block();
		} catch (e) {
			actual = e;
		}

		if (actual) {
			// we don't want to validate thrown error
			if (!expected) {
				ok = true;
			// expected is a regexp
			} else if (QUnit.objectType(expected) === "regexp") {
				ok = expected.test(actual);
			// expected is a constructor
			} else if (actual instanceof expected) {
				ok = true;
			// expected is a validation function which returns true is validation passed
			} else if (expected.call({}, actual) === true) {
				ok = true;
			}
		}

		QUnit.ok(ok, message);
	},

	start: function(count) {
		config.semaphore -= count || 1;
		if (config.semaphore > 0) {
			// don't start until equal number of stop-calls
			return;
		}
		if (config.semaphore < 0) {
			// ignore if start is called more often then stop
			config.semaphore = 0;
		}
		// A slight delay, to avoid any current callbacks
		if ( defined.setTimeout ) {
			window.setTimeout(function() {
				if (config.semaphore > 0) {
					return;
				}
				if ( config.timeout ) {
					clearTimeout(config.timeout);
				}

				config.blocking = false;
				process(true);
			}, 13);
		} else {
			config.blocking = false;
			process(true);
		}
	},

	stop: function(count) {
		config.semaphore += count || 1;
		config.blocking = true;

		if ( config.testTimeout && defined.setTimeout ) {
			clearTimeout(config.timeout);
			config.timeout = window.setTimeout(function() {
				QUnit.ok( false, "Test timed out" );
				config.semaphore = 1;
				QUnit.start();
			}, config.testTimeout);
		}
	}
};

//We want access to the constructor's prototype
(function() {
	function F(){};
	F.prototype = QUnit;
	QUnit = new F();
	//Make F QUnit's constructor so that we can add to the prototype later
	QUnit.constructor = F;
})();

// deprecated; still export them to window to provide clear error messages
// next step: remove entirely
QUnit.equals = function() {
	throw new Error("QUnit.equals has been deprecated since 2009 (e88049a0), use QUnit.equal instead");
};
QUnit.same = function() {
	throw new Error("QUnit.same has been deprecated since 2009 (e88049a0), use QUnit.deepEqual instead");
};

// Maintain internal state
var config = {
	// The queue of tests to run
	queue: [],

	// block until document ready
	blocking: true,

	// when enabled, show only failing tests
	// gets persisted through sessionStorage and can be changed in UI via checkbox
	hidepassed: false,

	// by default, run previously failed tests first
	// very useful in combination with "Hide passed tests" checked
	reorder: true,

	// by default, modify document.title when suite is done
	altertitle: true,

	urlConfig: ['noglobals', 'notrycatch'],

	//logging callback queues
	begin: [],
	done: [],
	log: [],
	testStart: [],
	testDone: [],
	moduleStart: [],
	moduleDone: []
};

// Load paramaters
(function() {
	var location = window.location || { search: "", protocol: "file:" },
		params = location.search.slice( 1 ).split( "&" ),
		length = params.length,
		urlParams = {},
		current;

	if ( params[ 0 ] ) {
		for ( var i = 0; i < length; i++ ) {
			current = params[ i ].split( "=" );
			current[ 0 ] = decodeURIComponent( current[ 0 ] );
			// allow just a key to turn on a flag, e.g., test.html?noglobals
			current[ 1 ] = current[ 1 ] ? decodeURIComponent( current[ 1 ] ) : true;
			urlParams[ current[ 0 ] ] = current[ 1 ];
		}
	}

	QUnit.urlParams = urlParams;
	config.filter = urlParams.filter;

	// Figure out if we're running the tests from a server or not
	QUnit.isLocal = !!(location.protocol === 'file:');
})();

// Expose the API as global variables, unless an 'exports'
// object exists, in that case we assume we're in CommonJS
if ( typeof exports === "undefined" || typeof require === "undefined" ) {
	extend(window, QUnit);
	window.QUnit = QUnit;
} else {
	module.exports = QUnit;
}

// define these after exposing globals to keep them in these QUnit namespace only
extend(QUnit, {
	config: config,

	// Initialize the configuration options
	init: function() {
		extend(config, {
			stats: { all: 0, bad: 0 },
			moduleStats: { all: 0, bad: 0 },
			started: +new Date,
			updateRate: 1000,
			blocking: false,
			autostart: true,
			autorun: false,
			filter: "",
			queue: [],
			semaphore: 0
		});

		var qunit = id( "qunit" );
		if ( qunit ) {
			qunit.innerHTML =
				'<h1 id="qunit-header">' + document.title + '</h1>' +
				'<h2 id="qunit-banner"></h2>' +
				'<div id="qunit-testrunner-toolbar"></div>' +
				'<h2 id="qunit-userAgent"></h2>' +
				'<ol id="qunit-tests"></ol>';
		}

		var tests = id( "qunit-tests" ),
			banner = id( "qunit-banner" ),
			result = id( "qunit-testresult" );

		if ( tests ) {
			tests.innerHTML = "";
		}

		if ( banner ) {
			banner.className = "";
		}

		if ( result ) {
			result.parentNode.removeChild( result );
		}

		if ( tests ) {
			result = document.createElement( "p" );
			result.id = "qunit-testresult";
			result.className = "result";
			tests.parentNode.insertBefore( result, tests );
			result.innerHTML = 'Running...<br/>&nbsp;';
		}
	},

	/**
	 * Resets the test setup. Useful for tests that modify the DOM.
	 *
	 * If jQuery is available, uses jQuery's replaceWith(), otherwise use replaceChild
	 */
	reset: function() {
		if ( window.jQuery ) {
			jQuery( "#qunit-fixture" ).replaceWith( config.fixture.cloneNode(true) );
		} else {
			var main = id( 'qunit-fixture' );
			if ( main ) {
				main.parentNode.replaceChild(config.fixture.cloneNode(true), main);
			}
		}
	},

	/**
	 * Trigger an event on an element.
	 *
	 * @example triggerEvent( document.body, "click" );
	 *
	 * @param DOMElement elem
	 * @param String type
	 */
	triggerEvent: function( elem, type, event ) {
		if ( document.createEvent ) {
			event = document.createEvent("MouseEvents");
			event.initMouseEvent(type, true, true, elem.ownerDocument.defaultView,
				0, 0, 0, 0, 0, false, false, false, false, 0, null);
			elem.dispatchEvent( event );

		} else if ( elem.fireEvent ) {
			elem.fireEvent("on"+type);
		}
	},

	// Safe object type checking
	is: function( type, obj ) {
		return QUnit.objectType( obj ) == type;
	},

	objectType: function( obj ) {
		if (typeof obj === "undefined") {
				return "undefined";

		// consider: typeof null === object
		}
		if (obj === null) {
				return "null";
		}

		var type = toString.call( obj ).match(/^\[object\s(.*)\]$/)[1] || '';

		switch (type) {
				case 'Number':
						if (isNaN(obj)) {
								return "nan";
						} else {
								return "number";
						}
				case 'String':
				case 'Boolean':
				case 'Array':
				case 'Date':
				case 'RegExp':
				case 'Function':
						return type.toLowerCase();
		}
		if (typeof obj === "object") {
				return "object";
		}
		return undefined;
	},

	push: function(result, actual, expected, message) {
		if (!config.current) {
			throw new Error("assertion outside test context, was " + sourceFromStacktrace());
		}
		var details = {
			result: result,
			message: message,
			actual: actual,
			expected: expected
		};

		message = escapeInnerText(message) || (result ? "okay" : "failed");
		message = '<span class="test-message">' + message + "</span>";
		var output = message;
		if (!result) {
			expected = escapeInnerText(QUnit.jsDump.parse(expected));
			actual = escapeInnerText(QUnit.jsDump.parse(actual));
			output += '<table><tr class="test-expected"><th>Expected: </th><td><pre>' + expected + '</pre></td></tr>';
			if (actual != expected) {
				output += '<tr class="test-actual"><th>Result: </th><td><pre>' + actual + '</pre></td></tr>';
				output += '<tr class="test-diff"><th>Diff: </th><td><pre>' + QUnit.diff(expected, actual) +'</pre></td></tr>';
			}
			var source = sourceFromStacktrace();
			if (source) {
				details.source = source;
				output += '<tr class="test-source"><th>Source: </th><td><pre>' + escapeInnerText(source) + '</pre></td></tr>';
			}
			output += "</table>";
		}

		runLoggingCallbacks( 'log', QUnit, details );

		config.current.assertions.push({
			result: !!result,
			message: output
		});
	},

	url: function( params ) {
		params = extend( extend( {}, QUnit.urlParams ), params );
		var querystring = "?",
			key;
		for ( key in params ) {
			if ( !hasOwn.call( params, key ) ) {
				continue;
			}
			querystring += encodeURIComponent( key ) + "=" +
				encodeURIComponent( params[ key ] ) + "&";
		}
		return window.location.pathname + querystring.slice( 0, -1 );
	},

	extend: extend,
	id: id,
	addEvent: addEvent
});

//QUnit.constructor is set to the empty F() above so that we can add to it's prototype later
//Doing this allows us to tell if the following methods have been overwritten on the actual
//QUnit object, which is a deprecated way of using the callbacks.
extend(QUnit.constructor.prototype, {
	// Logging callbacks; all receive a single argument with the listed properties
	// run test/logs.html for any related changes
	begin: registerLoggingCallback('begin'),
	// done: { failed, passed, total, runtime }
	done: registerLoggingCallback('done'),
	// log: { result, actual, expected, message }
	log: registerLoggingCallback('log'),
	// testStart: { name }
	testStart: registerLoggingCallback('testStart'),
	// testDone: { name, failed, passed, total }
	testDone: registerLoggingCallback('testDone'),
	// moduleStart: { name }
	moduleStart: registerLoggingCallback('moduleStart'),
	// moduleDone: { name, failed, passed, total }
	moduleDone: registerLoggingCallback('moduleDone')
});

if ( typeof document === "undefined" || document.readyState === "complete" ) {
	config.autorun = true;
}

QUnit.load = function() {
	runLoggingCallbacks( 'begin', QUnit, {} );

	// Initialize the config, saving the execution queue
	var oldconfig = extend({}, config);
	QUnit.init();
	extend(config, oldconfig);

	config.blocking = false;

	var urlConfigHtml = '', len = config.urlConfig.length;
	for ( var i = 0, val; i < len, val = config.urlConfig[i]; i++ ) {
		config[val] = QUnit.urlParams[val];
		urlConfigHtml += '<label><input name="' + val + '" type="checkbox"' + ( config[val] ? ' checked="checked"' : '' ) + '>' + val + '</label>';
	}

	var userAgent = id("qunit-userAgent");
	if ( userAgent ) {
		userAgent.innerHTML = navigator.userAgent;
	}
	var banner = id("qunit-header");
	if ( banner ) {
		banner.innerHTML = '<a href="' + QUnit.url({ filter: undefined }) + '"> ' + banner.innerHTML + '</a> ' + urlConfigHtml;
		addEvent( banner, "change", function( event ) {
			var params = {};
			params[ event.target.name ] = event.target.checked ? true : undefined;
			window.location = QUnit.url( params );
		});
	}

	var toolbar = id("qunit-testrunner-toolbar");
	if ( toolbar ) {
		var filter = document.createElement("input");
		filter.type = "checkbox";
		filter.id = "qunit-filter-pass";
		addEvent( filter, "click", function() {
			var ol = document.getElementById("qunit-tests");
			if ( filter.checked ) {
				ol.className = ol.className + " hidepass";
			} else {
				var tmp = " " + ol.className.replace( /[\n\t\r]/g, " " ) + " ";
				ol.className = tmp.replace(/ hidepass /, " ");
			}
			if ( defined.sessionStorage ) {
				if (filter.checked) {
					sessionStorage.setItem("qunit-filter-passed-tests", "true");
				} else {
					sessionStorage.removeItem("qunit-filter-passed-tests");
				}
			}
		});
		if ( config.hidepassed || defined.sessionStorage && sessionStorage.getItem("qunit-filter-passed-tests") ) {
			filter.checked = true;
			var ol = document.getElementById("qunit-tests");
			ol.className = ol.className + " hidepass";
		}
		toolbar.appendChild( filter );

		var label = document.createElement("label");
		label.setAttribute("for", "qunit-filter-pass");
		label.innerHTML = "Hide passed tests";
		toolbar.appendChild( label );
	}

	var main = id('qunit-fixture');
	if ( main ) {
		config.fixture = main.cloneNode(true);
	}

	if (config.autostart) {
		QUnit.start();
	}
};

addEvent(window, "load", QUnit.load);

// addEvent(window, "error") gives us a useless event object
window.onerror = function( message, file, line ) {
	if ( QUnit.config.current ) {
		ok( false, message + ", " + file + ":" + line );
	} else {
		test( "global failure", function() {
			ok( false, message + ", " + file + ":" + line );
		});
	}
};

function done() {
	config.autorun = true;

	// Log the last module results
	if ( config.currentModule ) {
		runLoggingCallbacks( 'moduleDone', QUnit, {
			name: config.currentModule,
			failed: config.moduleStats.bad,
			passed: config.moduleStats.all - config.moduleStats.bad,
			total: config.moduleStats.all
		} );
	}

	var banner = id("qunit-banner"),
		tests = id("qunit-tests"),
		runtime = +new Date - config.started,
		passed = config.stats.all - config.stats.bad,
		html = [
			'Tests completed in ',
			runtime,
			' milliseconds.<br/>',
			'<span class="passed">',
			passed,
			'</span> tests of <span class="total">',
			config.stats.all,
			'</span> passed, <span class="failed">',
			config.stats.bad,
			'</span> failed.'
		].join('');

	if ( banner ) {
		banner.className = (config.stats.bad ? "qunit-fail" : "qunit-pass");
	}

	if ( tests ) {
		id( "qunit-testresult" ).innerHTML = html;
	}

	if ( config.altertitle && typeof document !== "undefined" && document.title ) {
		// show ✖ for good, ✔ for bad suite result in title
		// use escape sequences in case file gets loaded with non-utf-8-charset
		document.title = [
			(config.stats.bad ? "\u2716" : "\u2714"),
			document.title.replace(/^[\u2714\u2716] /i, "")
		].join(" ");
	}

	// clear own sessionStorage items if all tests passed
	if ( config.reorder && defined.sessionStorage && config.stats.bad === 0 ) {
		for (var key in sessionStorage) {
			if (sessionStorage.hasOwnProperty(key) && key.indexOf("qunit-") === 0 ) {
				sessionStorage.removeItem(key);
			}
		}
	}

	runLoggingCallbacks( 'done', QUnit, {
		failed: config.stats.bad,
		passed: passed,
		total: config.stats.all,
		runtime: runtime
	} );
}

function validTest( name ) {
	var filter = config.filter,
		run = false;

	if ( !filter ) {
		return true;
	}

	var not = filter.charAt( 0 ) === "!";
	if ( not ) {
		filter = filter.slice( 1 );
	}

	if ( name.indexOf( filter ) !== -1 ) {
		return !not;
	}

	if ( not ) {
		run = true;
	}

	return run;
}

// so far supports only Firefox, Chrome and Opera (buggy)
// could be extended in the future to use something like https://github.com/csnover/TraceKit
function sourceFromStacktrace(offset) {
	offset = offset || 3;
	try {
		throw new Error();
	} catch ( e ) {
		if (e.stacktrace) {
			// Opera
			return e.stacktrace.split("\n")[offset + 3];
		} else if (e.stack) {
			// Firefox, Chrome
			var stack = e.stack.split("\n");
			if (/^error$/i.test(stack[0])) {
				stack.shift();
			}
			return stack[offset];
		} else if (e.sourceURL) {
			// Safari, PhantomJS
			// TODO sourceURL points at the 'throw new Error' line above, useless
			//return e.sourceURL + ":" + e.line;
		}
	}
}

function escapeInnerText(s) {
	if (!s) {
		return "";
	}
	s = s + "";
	return s.replace(/[\&<>]/g, function(s) {
		switch(s) {
			case "&": return "&amp;";
			case "<": return "&lt;";
			case ">": return "&gt;";
			default: return s;
		}
	});
}

function synchronize( callback, last ) {
	config.queue.push( callback );

	if ( config.autorun && !config.blocking ) {
		process(last);
	}
}

function process( last ) {
	var start = new Date().getTime();
	config.depth = config.depth ? config.depth + 1 : 1;

	while ( config.queue.length && !config.blocking ) {
		if ( !defined.setTimeout || config.updateRate <= 0 || ( ( new Date().getTime() - start ) < config.updateRate ) ) {
			config.queue.shift()();
		} else {
			window.setTimeout( function(){
				process( last );
			}, 13 );
			break;
		}
	}
	config.depth--;
	if ( last && !config.blocking && !config.queue.length && config.depth === 0 ) {
		done();
	}
}

function saveGlobal() {
	config.pollution = [];

	if ( config.noglobals ) {
		for ( var key in window ) {
			if ( !hasOwn.call( window, key ) ) {
				continue;
			}
			config.pollution.push( key );
		}
	}
}

function checkPollution( name ) {
	var old = config.pollution;
	saveGlobal();

	var newGlobals = diff( config.pollution, old );
	if ( newGlobals.length > 0 ) {
		ok( false, "Introduced global variable(s): " + newGlobals.join(", ") );
	}

	var deletedGlobals = diff( old, config.pollution );
	if ( deletedGlobals.length > 0 ) {
		ok( false, "Deleted global variable(s): " + deletedGlobals.join(", ") );
	}
}

// returns a new Array with the elements that are in a but not in b
function diff( a, b ) {
	var result = a.slice();
	for ( var i = 0; i < result.length; i++ ) {
		for ( var j = 0; j < b.length; j++ ) {
			if ( result[i] === b[j] ) {
				result.splice(i, 1);
				i--;
				break;
			}
		}
	}
	return result;
}

function fail(message, exception, callback) {
	if ( typeof console !== "undefined" && console.error && console.warn ) {
		console.error(message);
		console.error(exception);
		console.error(exception.stack);
		console.warn(callback.toString());

	} else if ( window.opera && opera.postError ) {
		opera.postError(message, exception, callback.toString);
	}
}

function extend(a, b) {
	for ( var prop in b ) {
		if ( b[prop] === undefined ) {
			delete a[prop];

		// Avoid "Member not found" error in IE8 caused by setting window.constructor
		} else if ( prop !== "constructor" || a !== window ) {
			a[prop] = b[prop];
		}
	}

	return a;
}

function addEvent(elem, type, fn) {
	if ( elem.addEventListener ) {
		elem.addEventListener( type, fn, false );
	} else if ( elem.attachEvent ) {
		elem.attachEvent( "on" + type, fn );
	} else {
		fn();
	}
}

function id(name) {
	return !!(typeof document !== "undefined" && document && document.getElementById) &&
		document.getElementById( name );
}

function registerLoggingCallback(key){
	return function(callback){
		config[key].push( callback );
	};
}

// Supports deprecated method of completely overwriting logging callbacks
function runLoggingCallbacks(key, scope, args) {
	//debugger;
	var callbacks;
	if ( QUnit.hasOwnProperty(key) ) {
		QUnit[key].call(scope, args);
	} else {
		callbacks = config[key];
		for( var i = 0; i < callbacks.length; i++ ) {
			callbacks[i].call( scope, args );
		}
	}
}

// Test for equality any JavaScript type.
// Author: Philippe Rathé <prathe@gmail.com>
QUnit.equiv = function () {

	var innerEquiv; // the real equiv function
	var callers = []; // stack to decide between skip/abort functions
	var parents = []; // stack to avoiding loops from circular referencing

	// Call the o related callback with the given arguments.
	function bindCallbacks(o, callbacks, args) {
		var prop = QUnit.objectType(o);
		if (prop) {
			if (QUnit.objectType(callbacks[prop]) === "function") {
				return callbacks[prop].apply(callbacks, args);
			} else {
				return callbacks[prop]; // or undefined
			}
		}
	}

	var getProto = Object.getPrototypeOf || function (obj) {
		return obj.__proto__;
	};

	var callbacks = function () {

		// for string, boolean, number and null
		function useStrictEquality(b, a) {
			if (b instanceof a.constructor || a instanceof b.constructor) {
				// to catch short annotaion VS 'new' annotation of a
				// declaration
				// e.g. var i = 1;
				// var j = new Number(1);
				return a == b;
			} else {
				return a === b;
			}
		}

		return {
			"string" : useStrictEquality,
			"boolean" : useStrictEquality,
			"number" : useStrictEquality,
			"null" : useStrictEquality,
			"undefined" : useStrictEquality,

			"nan" : function(b) {
				return isNaN(b);
			},

			"date" : function(b, a) {
				return QUnit.objectType(b) === "date"
						&& a.valueOf() === b.valueOf();
			},

			"regexp" : function(b, a) {
				return QUnit.objectType(b) === "regexp"
						&& a.source === b.source && // the regex itself
						a.global === b.global && // and its modifers
													// (gmi) ...
						a.ignoreCase === b.ignoreCase
						&& a.multiline === b.multiline;
			},

			// - skip when the property is a method of an instance (OOP)
			// - abort otherwise,
			// initial === would have catch identical references anyway
			"function" : function() {
				var caller = callers[callers.length - 1];
				return caller !== Object && typeof caller !== "undefined";
			},

			"array" : function(b, a) {
				var i, j, loop;
				var len;

				// b could be an object literal here
				if (!(QUnit.objectType(b) === "array")) {
					return false;
				}

				len = a.length;
				if (len !== b.length) { // safe and faster
					return false;
				}

				// track reference to avoid circular references
				parents.push(a);
				for (i = 0; i < len; i++) {
					loop = false;
					for (j = 0; j < parents.length; j++) {
						if (parents[j] === a[i]) {
							loop = true;// dont rewalk array
						}
					}
					if (!loop && !innerEquiv(a[i], b[i])) {
						parents.pop();
						return false;
					}
				}
				parents.pop();
				return true;
			},

			"object" : function(b, a) {
				var i, j, loop;
				var eq = true; // unless we can proove it
				var aProperties = [], bProperties = []; // collection of
														// strings

				// comparing constructors is more strict than using
				// instanceof
				if (a.constructor !== b.constructor) {
					// Allow objects with no prototype to be equivalent to
					// objects with Object as their constructor.
					if (!((getProto(a) === null && getProto(b) === Object.prototype) ||
						  (getProto(b) === null && getProto(a) === Object.prototype)))
					{
						return false;
					}
				}

				// stack constructor before traversing properties
				callers.push(a.constructor);
				// track reference to avoid circular references
				parents.push(a);

				for (i in a) { // be strict: don't ensures hasOwnProperty
								// and go deep
					loop = false;
					for (j = 0; j < parents.length; j++) {
						if (parents[j] === a[i])
							loop = true; // don't go down the same path
											// twice
					}
					aProperties.push(i); // collect a's properties

					if (!loop && !innerEquiv(a[i], b[i])) {
						eq = false;
						break;
					}
				}

				callers.pop(); // unstack, we are done
				parents.pop();

				for (i in b) {
					bProperties.push(i); // collect b's properties
				}

				// Ensures identical properties name
				return eq
						&& innerEquiv(aProperties.sort(), bProperties
								.sort());
			}
		};
	}();

	innerEquiv = function() { // can take multiple arguments
		var args = Array.prototype.slice.apply(arguments);
		if (args.length < 2) {
			return true; // end transition
		}

		return (function(a, b) {
			if (a === b) {
				return true; // catch the most you can
			} else if (a === null || b === null || typeof a === "undefined"
					|| typeof b === "undefined"
					|| QUnit.objectType(a) !== QUnit.objectType(b)) {
				return false; // don't lose time with error prone cases
			} else {
				return bindCallbacks(a, callbacks, [ b, a ]);
			}

			// apply transition with (1..n) arguments
		})(args[0], args[1])
				&& arguments.callee.apply(this, args.splice(1,
						args.length - 1));
	};

	return innerEquiv;

}();

/**
 * jsDump Copyright (c) 2008 Ariel Flesler - aflesler(at)gmail(dot)com |
 * http://flesler.blogspot.com Licensed under BSD
 * (http://www.opensource.org/licenses/bsd-license.php) Date: 5/15/2008
 *
 * @projectDescription Advanced and extensible data dumping for Javascript.
 * @version 1.0.0
 * @author Ariel Flesler
 * @link {http://flesler.blogspot.com/2008/05/jsdump-pretty-dump-of-any-javascript.html}
 */
QUnit.jsDump = (function() {
	function quote( str ) {
		return '"' + str.toString().replace(/"/g, '\\"') + '"';
	};
	function literal( o ) {
		return o + '';
	};
	function join( pre, arr, post ) {
		var s = jsDump.separator(),
			base = jsDump.indent(),
			inner = jsDump.indent(1);
		if ( arr.join )
			arr = arr.join( ',' + s + inner );
		if ( !arr )
			return pre + post;
		return [ pre, inner + arr, base + post ].join(s);
	};
	function array( arr, stack ) {
		var i = arr.length, ret = Array(i);
		this.up();
		while ( i-- )
			ret[i] = this.parse( arr[i] , undefined , stack);
		this.down();
		return join( '[', ret, ']' );
	};

	var reName = /^function (\w+)/;

	var jsDump = {
		parse:function( obj, type, stack ) { //type is used mostly internally, you can fix a (custom)type in advance
			stack = stack || [ ];
			var parser = this.parsers[ type || this.typeOf(obj) ];
			type = typeof parser;
			var inStack = inArray(obj, stack);
			if (inStack != -1) {
				return 'recursion('+(inStack - stack.length)+')';
			}
			//else
			if (type == 'function')  {
					stack.push(obj);
					var res = parser.call( this, obj, stack );
					stack.pop();
					return res;
			}
			// else
			return (type == 'string') ? parser : this.parsers.error;
		},
		typeOf:function( obj ) {
			var type;
			if ( obj === null ) {
				type = "null";
			} else if (typeof obj === "undefined") {
				type = "undefined";
			} else if (QUnit.is("RegExp", obj)) {
				type = "regexp";
			} else if (QUnit.is("Date", obj)) {
				type = "date";
			} else if (QUnit.is("Function", obj)) {
				type = "function";
			} else if (typeof obj.setInterval !== undefined && typeof obj.document !== "undefined" && typeof obj.nodeType === "undefined") {
				type = "window";
			} else if (obj.nodeType === 9) {
				type = "document";
			} else if (obj.nodeType) {
				type = "node";
			} else if (
				// native arrays
				toString.call( obj ) === "[object Array]" ||
				// NodeList objects
				( typeof obj.length === "number" && typeof obj.item !== "undefined" && ( obj.length ? obj.item(0) === obj[0] : ( obj.item( 0 ) === null && typeof obj[0] === "undefined" ) ) )
			) {
				type = "array";
			} else {
				type = typeof obj;
			}
			return type;
		},
		separator:function() {
			return this.multiline ?	this.HTML ? '<br />' : '\n' : this.HTML ? '&nbsp;' : ' ';
		},
		indent:function( extra ) {// extra can be a number, shortcut for increasing-calling-decreasing
			if ( !this.multiline )
				return '';
			var chr = this.indentChar;
			if ( this.HTML )
				chr = chr.replace(/\t/g,'   ').replace(/ /g,'&nbsp;');
			return Array( this._depth_ + (extra||0) ).join(chr);
		},
		up:function( a ) {
			this._depth_ += a || 1;
		},
		down:function( a ) {
			this._depth_ -= a || 1;
		},
		setParser:function( name, parser ) {
			this.parsers[name] = parser;
		},
		// The next 3 are exposed so you can use them
		quote:quote,
		literal:literal,
		join:join,
		//
		_depth_: 1,
		// This is the list of parsers, to modify them, use jsDump.setParser
		parsers:{
			window: '[Window]',
			document: '[Document]',
			error:'[ERROR]', //when no parser is found, shouldn't happen
			unknown: '[Unknown]',
			'null':'null',
			'undefined':'undefined',
			'function':function( fn ) {
				var ret = 'function',
					name = 'name' in fn ? fn.name : (reName.exec(fn)||[])[1];//functions never have name in IE
				if ( name )
					ret += ' ' + name;
				ret += '(';

				ret = [ ret, QUnit.jsDump.parse( fn, 'functionArgs' ), '){'].join('');
				return join( ret, QUnit.jsDump.parse(fn,'functionCode'), '}' );
			},
			array: array,
			nodelist: array,
			arguments: array,
			object:function( map, stack ) {
				var ret = [ ];
				QUnit.jsDump.up();
				for ( var key in map ) {
					var val = map[key];
					ret.push( QUnit.jsDump.parse(key,'key') + ': ' + QUnit.jsDump.parse(val, undefined, stack));
				}
				QUnit.jsDump.down();
				return join( '{', ret, '}' );
			},
			node:function( node ) {
				var open = QUnit.jsDump.HTML ? '&lt;' : '<',
					close = QUnit.jsDump.HTML ? '&gt;' : '>';

				var tag = node.nodeName.toLowerCase(),
					ret = open + tag;

				for ( var a in QUnit.jsDump.DOMAttrs ) {
					var val = node[QUnit.jsDump.DOMAttrs[a]];
					if ( val )
						ret += ' ' + a + '=' + QUnit.jsDump.parse( val, 'attribute' );
				}
				return ret + close + open + '/' + tag + close;
			},
			functionArgs:function( fn ) {//function calls it internally, it's the arguments part of the function
				var l = fn.length;
				if ( !l ) return '';

				var args = Array(l);
				while ( l-- )
					args[l] = String.fromCharCode(97+l);//97 is 'a'
				return ' ' + args.join(', ') + ' ';
			},
			key:quote, //object calls it internally, the key part of an item in a map
			functionCode:'[code]', //function calls it internally, it's the content of the function
			attribute:quote, //node calls it internally, it's an html attribute value
			string:quote,
			date:quote,
			regexp:literal, //regex
			number:literal,
			'boolean':literal
		},
		DOMAttrs:{//attributes to dump from nodes, name=>realName
			id:'id',
			name:'name',
			'class':'className'
		},
		HTML:false,//if true, entities are escaped ( <, >, \t, space and \n )
		indentChar:'  ',//indentation unit
		multiline:true //if true, items in a collection, are separated by a \n, else just a space.
	};

	return jsDump;
})();

// from Sizzle.js
function getText( elems ) {
	var ret = "", elem;

	for ( var i = 0; elems[i]; i++ ) {
		elem = elems[i];

		// Get the text from text nodes and CDATA nodes
		if ( elem.nodeType === 3 || elem.nodeType === 4 ) {
			ret += elem.nodeValue;

		// Traverse everything else, except comment nodes
		} else if ( elem.nodeType !== 8 ) {
			ret += getText( elem.childNodes );
		}
	}

	return ret;
};

//from jquery.js
function inArray( elem, array ) {
	if ( array.indexOf ) {
		return array.indexOf( elem );
	}

	for ( var i = 0, length = array.length; i < length; i++ ) {
		if ( array[ i ] === elem ) {
			return i;
		}
	}

	return -1;
}

/*
 * Javascript Diff Algorithm
 *  By John Resig (http://ejohn.org/)
 *  Modified by Chu Alan "sprite"
 *
 * Released under the MIT license.
 *
 * More Info:
 *  http://ejohn.org/projects/javascript-diff-algorithm/
 *
 * Usage: QUnit.diff(expected, actual)
 *
 * QUnit.diff("the quick brown fox jumped over", "the quick fox jumps over") == "the  quick <del>brown </del> fox <del>jumped </del><ins>jumps </ins> over"
 */
QUnit.diff = (function() {
	function diff(o, n) {
		var ns = {};
		var os = {};

		for (var i = 0; i < n.length; i++) {
			if (ns[n[i]] == null)
				ns[n[i]] = {
					rows: [],
					o: null
				};
			ns[n[i]].rows.push(i);
		}

		for (var i = 0; i < o.length; i++) {
			if (os[o[i]] == null)
				os[o[i]] = {
					rows: [],
					n: null
				};
			os[o[i]].rows.push(i);
		}

		for (var i in ns) {
			if ( !hasOwn.call( ns, i ) ) {
				continue;
			}
			if (ns[i].rows.length == 1 && typeof(os[i]) != "undefined" && os[i].rows.length == 1) {
				n[ns[i].rows[0]] = {
					text: n[ns[i].rows[0]],
					row: os[i].rows[0]
				};
				o[os[i].rows[0]] = {
					text: o[os[i].rows[0]],
					row: ns[i].rows[0]
				};
			}
		}

		for (var i = 0; i < n.length - 1; i++) {
			if (n[i].text != null && n[i + 1].text == null && n[i].row + 1 < o.length && o[n[i].row + 1].text == null &&
			n[i + 1] == o[n[i].row + 1]) {
				n[i + 1] = {
					text: n[i + 1],
					row: n[i].row + 1
				};
				o[n[i].row + 1] = {
					text: o[n[i].row + 1],
					row: i + 1
				};
			}
		}

		for (var i = n.length - 1; i > 0; i--) {
			if (n[i].text != null && n[i - 1].text == null && n[i].row > 0 && o[n[i].row - 1].text == null &&
			n[i - 1] == o[n[i].row - 1]) {
				n[i - 1] = {
					text: n[i - 1],
					row: n[i].row - 1
				};
				o[n[i].row - 1] = {
					text: o[n[i].row - 1],
					row: i - 1
				};
			}
		}

		return {
			o: o,
			n: n
		};
	}

	return function(o, n) {
		o = o.replace(/\s+$/, '');
		n = n.replace(/\s+$/, '');
		var out = diff(o == "" ? [] : o.split(/\s+/), n == "" ? [] : n.split(/\s+/));

		var str = "";

		var oSpace = o.match(/\s+/g);
		if (oSpace == null) {
			oSpace = [" "];
		}
		else {
			oSpace.push(" ");
		}
		var nSpace = n.match(/\s+/g);
		if (nSpace == null) {
			nSpace = [" "];
		}
		else {
			nSpace.push(" ");
		}

		if (out.n.length == 0) {
			for (var i = 0; i < out.o.length; i++) {
				str += '<del>' + out.o[i] + oSpace[i] + "</del>";
			}
		}
		else {
			if (out.n[0].text == null) {
				for (n = 0; n < out.o.length && out.o[n].text == null; n++) {
					str += '<del>' + out.o[n] + oSpace[n] + "</del>";
				}
			}

			for (var i = 0; i < out.n.length; i++) {
				if (out.n[i].text == null) {
					str += '<ins>' + out.n[i] + nSpace[i] + "</ins>";
				}
				else {
					var pre = "";

					for (n = out.n[i].row + 1; n < out.o.length && out.o[n].text == null; n++) {
						pre += '<del>' + out.o[n] + oSpace[n] + "</del>";
					}
					str += " " + out.n[i].text + nSpace[i] + pre;
				}
			}
		}

		return str;
	};
})();

// get at whatever the global object is, like window in browsers
})( (function() {return this}).call() );
```

  

Documentation generated by JSDoc 3.4.1 on Mon Oct 03 2016 12:37:32 GMT+0100 (BST) using the docdash theme.
